# Comparison of Two Rapid Assays for the Detection of *BRAF* V600 Mutations in Metastatic Melanoma including Positive Sentinel Lymph Nodes

**DOI:** 10.3390/diagnostics12030751

**Published:** 2022-03-19

**Authors:** Elodie Long-Mira, Alexandra Picard-Gauci, Sandra Lassalle, Véronique Hofman, Salomé Lalvée, Virginie Tanga, Katia Zahaf, Christelle Bonnetaud, Virginie Lespinet, Olivier Camuzard, Henri Montaudié, Gilles Poissonnet, Thierry Passeron, Marius Ilié, Paul Hofman

**Affiliations:** 1 CHU Nice, FHU OncoAge, Laboratory of Clinical and Experimental Pathology, Pasteur Hospital, Université Côte d’Azur, 06000 Nice, France; long.mira.e@chu-nice.fr (E.L.-M.); lassalle.s@chu-nice.fr (S.L.); hofman.v@chu-nice.fr (V.H.); lalvee.s@chu-nice.fr (S.L.); tanga.v@chu-nice.fr (V.T.); zahaf.k@chu-nice.fr (K.Z.); bonnetaud.c@chu-nice.fr (C.B.); lespinet.v@chu-nice.fr (V.L.); 2 CNRS, INSERM, IRCAN, Team 4, FHU OncoAge, Centre Antoine Lacassagne, Université Côte d’Azur, 06100 Nice, France; 3 CHU Nice, FHU OncoAge, Hospital-Integrated Biobank (BB-0033-00025), Université Côte d’Azur, 06000 Nice, France; 4 CHU Nice, Department of Dermatology, Archet Hospital, Université Côte d’Azur, 06200 Nice, France; picard-gauci.a@chu-nice.fr (A.P.-G.); montaudie.h@chu-nice.fr (H.M.); passeron.t@chu-nice.fr (T.P.); 5 Department of Plastic and Reconstructive Surgery, Institut Universitaire Locomoteur & Sport (iULS), Pasteur Hospital, Université Côte d’Azur, 06000 Nice, France; camuzard.o@chu-nice.fr; 6 Centre Antoine Lacassagne, Cervico-Facial Surgery Department, 06100 Nice, France; gilles.poissonnet@nice.unicancer.fr

**Keywords:** metastatic melanoma, *BRAF*, RT-PCR, sentinel lymph node, immunohistochemistry

## Abstract

Testing for the *BRAF* mutation is mandatory for the management of patients with locally advanced or metastatic melanoma. Molecular analysis based on DNA sequencing remains the gold-standard method for the screening of the different *BRAF* mutations. These methods must be rapid, sensitive, and specific enough to allow optimal therapeutic management in daily practice and also to include patients in clinical trials. Here, we compared the Idylla *BRAF* Mutation Test and the anti-*BRAF V600E* (clone VE1) immunohistochemistry (IHC) in 90 melanoma samples, with a focus on a challenging cohort of 32 positive sentinel lymph nodes. The *BRAF* status was assessed with both methods independently of the percentage of tumor cells. The concordance rate was calculated excluding both non-contributory analyses and *BRAF* *V600K/R/M* mutants due to the specific V600E-IHC test design. The incidence of the *BRAF* *V600E* mutation was 33% with both *BRAF* Idylla and *BRAF* IHC. The agreement rate was 91% (72/79). Although the agreement rate was high, we suggest that the use of IHC is more suitable for rapid *BRAF* testing on sentinel lymph node biopsies when associated with a low percentage and scattered tumor cells, which gave a high risk of non-contributory analysis and/or false negative results with the Idylla^TM^ *BRAF* Mutation Test.

## 1. Introduction

Melanoma is an aggressive skin cancer with an increasing incidence worldwide [1,2]. Histopathological examination of melanoma is the gold standard for providing prognostic information according to the American Joint Committee on Cancer (AJCC) classification [3]. However, some early-stage patients still recur or metastasize [4]. Sentinel lymph node (SLN) examination helps for better staging procedure. Therefore, SLN biopsy has to be performed as a staging procedure and treatment decision in patients with tumor thickness (Breslow) ≥1 mm or ≥0.8 mm with additional risk factors [4,5]. Moreover, this procedure is now mandatory for the new adjuvant option, allowing the inclusion of patients in different clinical trials [5]. 

In the last few years, new therapeutic options such as molecular-targeted therapies and immunotherapy have improved the outcomes of patients with metastatic melanoma [6]. In this regard, screening for predictive biomarkers is mandatory for the management of advanced or metastatic cutaneous melanoma, but since the establishment of different adjuvant and neoadjuvant treatments in primary tumors (pT2b-pT4) or SLN, the indications for *BRAF* testing are increasing considerably [3,5]. In particular, there is an essential need to perform this testing within a short turnaround time on increasingly smaller-sized specimens. 

*BRAF V600* mutations are oncogenic drivers of cutaneous melanoma and are detected in approximately 40% of cases [7,8]. The most frequent mutation is a substitution of glutamic acid for valine at codon 600 (p.*V600E*), occurring in around 75% of the cases. Various methods for the detection of the *BRAF V600E* mutation have been developed, in particular targeted molecular methods based on the analysis of DNA or protein expression evaluated with immunohistochemistry (IHC) on formalin-fixed and paraffin embedded (FFPE) tissue sections or cytological specimens [9,10]. The *BRAF V600E* mutation-specific antibody (clone VE1) is a monoclonal antibody specific for the mutated *V600E* epitope [11,12]. This anti-BRAF V600E IHC can be easily set up in a pathology laboratory and used routinely in accordance with quality control/assurance and accreditation procedures [13]. DNA-based analyses usually require a dedicated space to set up the equipment, to avoid contamination, and need highly qualified staff and a mastered turnaround time [14]. The Idylla^TM^ platform (Biocartis, Mechelen, Belgium) is a fully automated PCR-based system designed to be easy to implement and use in a pathology laboratory. It requires little space, avoids contamination, and necessitates only a short handling time. Both the *BRAF* Idylla^TM^ Mutation test and the VE1 IHC are fast, sensitive, and specific methods, while requiring limited tumor material.

We conducted a comparative study of the performance of two validated methods set up at the Laboratory of Clinical and Experimental Pathology (LCEP) (Nice, France), the *BRAF* VE1 IHC (accredited according to the ISO 15189 norm: www.cofrac.fr, accessed on 19 February 2022) and the *BRAF* Idylla Mutation test, on 90 melanoma FFPE samples, including 32 positive sentinel lymph nodes (SLN). In addition, we reviewed the literature on the performance of the *BRAF* Idylla test for melanoma.

## 2. Materials and Methods

### 2.1. Samples

Fifty-eight unselected FFPE melanoma tissue samples from the LCEP, Nice University Hospital, were included consecutively between January 2018 and January 2019. In addition, 32 positive SLN were included retrospectively. The percentage of tumor cells (%TC) in melanoma samples was evaluated independently by four senior pathologists (ELM, MI, SL, and VH) on routine hematoxylin-eosin-safran (HES) stained tissue sections, according to the procedures of the French association for quality assurance in pathology (AFAQAP) [15]. All patients gave their informed consent, and the study was conducted according to the Helsinki guidelines.

### 2.2. Assessment of the BRAF Mutation Status with the Idylla ^TM^ Method

The *BRAF* mutation status was assessed routinely at the LCEP on the Idylla^TM^ platform (Biocartis, Mechelen, Belgium). The CE-IVD Idylla^TM^ *BRAF* Mutation Test is a fully automated cartridge, ready-to-use, with all reagents on-board to remove paraffin, lyse the sample, extract, and amplify DNA. The tumor area was macrodissected and transferred to the cartridge (as per the manufacturer’s instructions). A systematic HES control stain after each macrodissection ensured that the tumor material was properly selected. After a 90 min run and less than 2 min hands-on time, the final report was directly available on the console. The value of Cq (quantification cycle) was determined by different parameters related to the amplification curves generated by the Idylla^TM^ Explore software. The difference between the Cq of the internal control and the variant (or ΔCq) identified the presence or absence of a mutation with a high sensitivity, down to 1% of mutant allele (according to manufacturer’s instructions) [16,17]. The Idylla^TM^ *BRAF* Mutation Test can detect 7 mutations in the *BRAF* gene (Table 1). As the real time PCR uses allele specific primers separated into two chambers, the result is given as “V600E/E2/D Mutation” or “V600K/R/M Mutation” or “Wild Type”. 

### 2.3. Assessment of the BRAF V600E Status with Immunohistochemistry

IHC was performed on the same FFPE block, after molecular analysis, with the *BRAF V600E* mutation-specific antibody (mouse monoclonal, clone VE1, prediluted, 16-min incubation, Roche Ventana, Tucson, AZ, USA) as previously described at the LCEP [18]. The protocol was applied to 3 μm tissue sections with an automated immunostainer (Ventana Benchmark Ultra; Ventana Medical Systems, Tucson, AZ, USA) using the OptiView DAB IHC Detection Kit (Ventana). All slides were reviewed independently of the clinicopathological parameters and the results of the molecular biology by one pathologist (ELM) for the purpose of the study, as reported previously [13]. 

### 2.4. Literature Search

An electronic search of the Medline database (using PubMed as search interface) was performed. We used the following medical terms and headings for the search: “BRAF”, “melanoma”, “Idylla” or “RT-PCR” and “IHC”, “V600E,” “Idylla”, or “RT-PCR”. We screened all studies comparing IHC with Idylla for the detection of *BRAF V600E* and Idylla with other genetic analyses for melanoma patients, published up to April 2021.

## 3. Results

### 3.1. Samples

We studied 90 melanoma samples, including 77 metastases and 13 advanced-stage primary tumors. Metastatic sites included the lymph node (n = 54), the subcutaneous area (n = 14), the lung (n = 7) and miscellaneous sites (n = 2). 60% (32/54) of the lymph node metastases were SLN. The SLN tumor burden was evaluated according to the combined Rotterdam [19] and Dewar criteria [20]. The tumor size, evaluated according to Rotterdam criteria, was <0.1 mm for 1/32 (3%) of cases, between 0.1–1 mm for 14/32 (43%) of cases, and greater than 1 mm for 17/32 (53%) of cases with a mean of 3.69 mm [0.55–14]. The microanatomical localization, evaluated according to Dewar criteria, was combined (10/32), sub capsular (8/32), multifocal (6/32), parenchymal (6/32), and extensive (2/32) (Table 2).

### 3.2. Molecular Testing

The Idylla molecular analysis found 57/85 (67%) wt-*BRAF V600*, 28/85 (33%) mutant-*BRAF* and 5/90 (5.5%) non-contributive cases due to insufficient DNA input (Table 2). All of the failed results were observed in SLN (5/32; 16%). In these cases, the metastatic involvement was multifocal or parenchymal according to Dewar criteria. The tumor size ranged from approximately 10 TC (Rotterdam < 0.1 mm) to 3 mm (mean 1.16 mm [<0.1–3 mm]) and the macrodissected surface area was from 10 to 30 mm^2^.

### 3.3. Immunohistochemistry

IHC analysis showed 29/90 (33%) *BRAF V600E* positive results and 59/90 (65%) negative results. All *V600K/R/M* mutated cases were VE1 IHC negative. VE1 IHC was not contributive (no detectable tumor cells) in 2/90 (2%) cases corresponding to SLN, the tumor burden of which was evaluated at less than 0.1 mm and between 0.1–1 mm. These two cases were also not contributive using the *BRAF* Idylla^TM^ Mutation Test (due to an insufficient amount of DNA).

### 3.4. Performance Comparison of Both Methods

**Figure 1:**
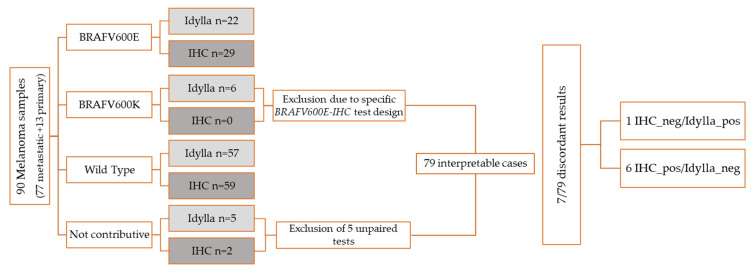
Flowcharts of the results of testing for *BRAF* with IHC and molecular method.

The overall agreement between IHC and Idylla for the assessment of the *BRAF* status was calculated on 79 cases, excluding the non-contributory cases and the *BRAF V600K/R/M* mutated results due to the use of the specific *BRAF V600E*-IHC test (Figure 1).

After excluding the unpaired cases (IHC or Idylla alone) and the *BRAF V600K/R/M* results due to the specific *BRAF V600E-IHC* test design, 79 cases were included in the statistical analysis.

According to these criteria, 71/79 (90%) of the cases had concordant results. Among the discordant results, we observed one Idylla *BRAF V600E/E2/D* with a negative VE1 IHC and seven Idylla wt-*BRAF V600* with a positive VE1 IHC. All VE1 IHC were reviewed as follows: four had 5%TC and the others had equal or less than 1%TC. The VE1 IHC was repeated for these eight cases, and one sample was then reclassified as negative due to non-specific melanophage staining. 

After reclassification of the discordant VE1 IHC sample, the agreement was 72/79 (91%). The concordance rate was even higher for 57/58 (98%) when only the “non-sentinel” tissue specimens were analyzed. Interestingly, while the supplier recommended a minimum amount of 50% TC for the Idylla *BRAF* assay, we did not detect any false negative results for the *BRAF V600E* mutation compared to VE1 IHC when the threshold was >10% TC.

Among the discordant samples, all the six positive VE1 IHC cases were analyzed with a molecular method on another FFPE block (metastasis or primitive), resulting in a *BRAF V600* mutation in 100% of cases (Table 3). 

**Figure 2:**
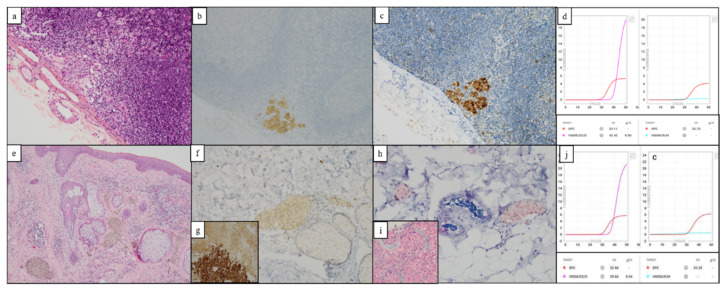
Example of discordant results. (**a**–**d**), a micro-metastatic sentinel lymph node that expressed *BRAF V600E* when analyzed by IHC and was negative with the *BRAF* Idylla test despite delayed amplification. (**e**–**g**), a subcutaneous melanoma metastasis considered negative for IHC *BRAF V600E* when compared to the control. (**g**) (some background noise due to intracytoplasmic pigment), when stained with a red chromogen the interpretation remains equivocal (**h**,**i**) and positive with the Idylla method. (**a**,**e**) HES stain ×20; (**b**) Melan A IHC ×20 (Clone A103, Roche Ventana); (**c**,**f**) *BRAF V600E* IHC ×20 (Clone VE1, Roche Ventana); (**g**) *BRAF V600E* IHC ×40 external control showing viable tumor cells and melanophages; (**h**) *BRAF V600E* IHC ×20 (Clone VE1, Roche Ventana—Red detection kit); (**i**) *BRAF V600E* IHC ×40 external control viable tumor cells with red chromogen; (**d**,**j**) Amplification curves with the Idylla Explore tool.

Because of faint labelling of tumor cells, the discordant sample—VE1 IHC negative and *BRAF V600E/E2/D* Idylla positive—was tested again with *BRAF* VE1 IHC using a red chromogen (UltraView Universal Alkaline Phosphatase Red Detection Kit, Roche). Interpretation remained equivocal due to persistent weak cytoplasmic staining. NGS analysis of the same sample with the Ion AmpliSeq™ Cancer Hotspot Panel V2 using the Ion AmpliSeq™ Library Kit™ (Ion Genestudio™ S5 Thermo Fisher Scientific, Illkirch-Graffenstaden, France) was *BRAF* wild type, whereas we expected to observe a *BRAF V600D* mutation (not detected with the VE1 antibody). This discrepancy between these two molecular biology methods may be related to a difference in the sensitivity threshold (5% sensitivity for the NGS method vs. 1% for Idylla). In addition, in this case, a new subcutaneous metastasis collected after treatment with a BRAF inhibitor was analyzed by the three methods (IHC, NGS, and Idylla), and did not reveal a *BRAF V600E* mutation. Of note, the *BRAF V600* mutation status is usually consistent between primary melanomas and matched metastases, even after targeted therapy [21,22,23]. Overall, a false positive result with the Idylla method cannot be ruled out (Figure 2).

### 3.5. Interpretation of the Results

According to the sensitivity threshold of the Idylla method, 6/85 (7%) of cases were at risk of false negative results using a molecular biology approach (defined as a %TC ≤ 1%), all in the SLN cohort. Examination of these cases with the Idylla Explore tool revealed that 3/6 showed late amplification curves with delayed Cq and a ΔCq ranging from 9.9 to 10.98. In addition, 3/6 cases could be tested on other metastases or on the primary samples with the same Idylla^TM^ method. The results showed 2/4 mutated cases and 2/4 WT cases, i.e., a proven risk of a false negative in 50% of the cases when the percentage of tumor cells is 1% or less (Table 4). 

## 4. Discussion

We compared two biomarker-screening methods, DNA- and protein-based, to assess the *BRAF* mutation status in a cohort of 90 melanoma samples enriched in SLN with a low TC content. Our results showed a high agreement between these two methods, and the incidence of the *BRAF V600E* mutation reported in our study is representative of that published in the literature [24]. 

In total, 16 studies [16,17,25,26,27,28,29,30,31,32,33,34,35,36,37,38] have already reported a comparison between the Idylla method and other molecular reference methods for the assessment of the *BRAF V600E* mutation in melanoma patients at the time of the publication (Table 5). Most of them used the CE-IVD Idylla^TM^ *BRAF* Mutation Test with a concordance rate ranging from 96.2% to 100% when compared with NGS, Sanger sequencing, pyrosequencing, or digital PCR, confirming that the test is reliable.

In addition, nine previous studies reported results using the RT-PCR Idylla method and VE1 IHC for the diagnostic of *BRAF* mutations [28,29,33,35,39,40,41,42,43]. Among these, four focused on melanoma samples [28,29,33,35]. the others included central nervous system tumors, colorectal cancers, ovarian tumors, hairy cell leukemia, and salivary gland tumors [39,40,41,42,43] (Table 6). Among melanoma samples, the concordance rate was high (89–100%), with good sensitivity (82.3–94%) and specificity (95–100%). The discordant cases were mostly due to inadequate preanalytical treatment (as decalcification) and reflect the difficulties of interpretation of VE1 IHC, especially due to the use of a chromogen not suitable for a melanocytic pathology. 

Unlike previous studies, the current study includes positive SLN biopsies with microscopic metastases where the estimation of TC for molecular analysis is challenging and responsible for an increased risk of false negatives with molecular testing methods [44,45,46,47]. Since new adjuvant treatments in completely resected stage II-IV melanoma have demonstrated a significant impact on relapse-free survival and overall survival in patients with *BRAFV600*-mutant melanoma [48,49], the early detection of a *BRAF V600* mutation in primary tumors (pT2b-pT4) or SLN is crucial, even at an early tumor stage [4]. These new therapeutic strategies underscore the value of testing positive SLN regardless of their tumor burden. The risk of false negative could be reduced with a macrodissection step to help minimize interobserver variation when evaluating the TC content and enrich the sample with TC for molecular biology. However, it is difficult to achieve in SLN because the tumor surface area is very restricted (i.e., multifocal or dispersed distribution—not compatible with the minimum surface area to be analyzed with Idylla). Thus, macrodissection on glass slides is most often responsible for failure due to insufficient DNA input, and macrodissection on paraffin blocks gives a high degree of variation in the cellularity due to fleeting micrometastases. The results, at risk of false negatives, must be reported and a supplementary analysis must be performed on another FFPE sample; the only alternative for these small-stage tumors remains, therefore, the analysis of the primary site. This raises the problem of the tumor heterogeneity reported in different studies [50,51,52], although sometimes not very comparable because of the different molecular analysis tests that are performed (NGS versus targeted sequencing, sensitivity threshold...) [53,54,55]. Other studies suggest that the *BRAF* mutation status of the primary tumor is retained in metastases [56,57] and that primary and/or metastatic tissue can be used for routine mutational analysis provided that sufficient TC content is available. Thus, the consistency of *BRAF* mutations among primary and metastatic tumors is still being debated. Finally, the question of inter-tumor heterogeneity also exists in the presence of several synchronous primary tumors, which sometimes happens in sun-exposed patients [57]. 

The Idylla^TM^ *BRAF* Mutation Test allows the detection of the actionable *BRAF V600E/D/K/R/M* mutations with a low amount of material and has good sensitivity, but is not able to make a distinction between them, which can be an obstacle to predicting the therapeutic response [58,59]. Another limitation is the impossibility of collecting DNA from the cartridge for further analysis, such as NGS. Moreover, in several cases of SLN, it cannot be performed because it cannot meet the supplier’s recommendations (especially regarding the tumor sample size and the % of TC), although our results suggest that 10% TC is appropriate for the detection of *BRAF V600E* (Table 7). In the present study, the 5/90 cases that remained unamplified with Idylla contained all had low melanin content. Melanin has been recognized as a PCR inhibitor [60], but it seems to have little impact on the Idylla technology [37]. Here, the lack of amplification was related rather to an insufficient intake of tissue.

At LCEP (Nice, France), we apply the established European Organisation for Research and Treatment of Cancer (EORTC) protocol for SLN [61,62], which saves unstained slides that can be used for complementary IHC, in particular VE1 IHC. With this approach, *BRAF* evaluation was possible on all but two samples with VE1 IHC, without a risk of false negative results as long as the pre-analytical steps are mastered. The major advantage of VE1 IHC is that it is highly suitable for small specimens, even at a single cell level, and uses minimal tissue. In addition, it is easy to implement and cost-effective. Nevertheless, its disadvantages lie in the fact that it only detects the *BRAF V600E* mutant. In addition, it can be more easily subjected to pre-analytical variations, inducing a risk of false negative results and some pitfalls associated with background noise or an alteration in the extent, distribution, and intensity of the staining [35,63]. The pathologist must also be well trained in the interpretation of VE1 IHC in order to avoid the risk of false positive results. A positive *BRAF V600E* IHC is most often strong and diffuse throughout the tumor cells [11,13,64]. Weak or melanophage related staining should be interpreted carefully as equivocal or uninterpretable and requires control with molecular biology. In pigmented tumors with extensive melanin an AEC–type (3-amino-9 ethylcarbazole) red chromogen can also be used instead of the DAB (3,3′-Diaminobenzidine) brown chromogen to help with interpretation. The interpretation of a negative VE1 IHC may also be challenging as it is necessary to ensure that TC are present in the IHC section, especially in SLN. In any case, a negative IHC must be confirmed by molecular analysis to avoid the risk (even low) of false negative IHC results and in order to detect other *BRAF V600* mutations aside from the *BRAF V600E,* notably the *BRAF V600K* mutation, for which a therapeutic response is also observed with *BRAF* and MEK inhibitors [48,65,66,67]. Rare *BRAF* mutations on codon 597 or 601, also reported to be moderately sensitive to *BRAF* and MEK inhibitors, are not detected with an allele-specific method or IHC. Next-generation sequencing can overcome this issue, and results can be discussed on specific molecular tumor boards.

SLN testing represents a major burden for pathology laboratories. Interestingly, the Merlin test (SkylineDx), recently developed on the Biocartis Idylla™ molecular diagnostic platform, aims to predict patients at low risk for lymph node metastasis [68,69]. This test, which has yet to undergo clinical validation, would reduce negative SLN biopsies, which, in addition to being a benefit to the patient, would reduce the laboratory workload and allow for a comprehensive review of the remaining SLNs. 

## 5. Conclusions

This study demonstrated that the VE1 IHC and *BRAF* Idylla methods are accurate and highly correlated to the detection of the *BRAF V600E* mutation in melanoma. IHC is often promoted as a prescreening tool, but it definitely suits small sample sizes with few TC. For metastatic SLN, we first recommend the use of VE1 IHC, which is easy to perform using the EORTC protocol. In the case of a negative VE1 IHC, a molecular analysis can be achieved immediately on the same sample if the percentage of tumor cells and tumor surface areas are sufficient to obtain enough extracted tumor DNA. The risk of false negative results can be prevented with the selection of an adequate specimen (cellularity—melanin load—mastered fixative condition—decalcification) and the use of a molecular assay with high sensitivity. Alternatively, in the case of negative results due to a very low number of tumor cells, a liquid biopsy can now open up new promises to evaluate the *BRAF* status in these patients [26,30,70].

## Figures and Tables

**Table 1:** Overview of mutations detected with the Idylla *BRAF* Assay (Idylla^TM^ platform—Biocartis, Mechelen, Belgium).

Exon	Results Given with Idylla	Mutation Detected
15	*V600E/E2/D*	c.1799T > A
		c.1799_1800TG > AA
		c.1799_1800TG > AT
		c.1799_1800TG > AC
	*V600K/R/M*	c.1798_1799GT > AA
		c.1798_1799GT > AG
		c.1798G > A
	Wild Type	c.1799T

**Table 2:** (A) Clinical and pathological characteristics of the 90 melanoma patients included in the study. (B) Focus on the metastatic SLN samples.

**(A)**		
**Clinical and Pathological Characteristics**		**N (%)**
		**90 (100)**
Age at diagnostic	Median	63
	Range	10–93
Gender	Male	42 (47)
	Female	48 (53)
Tissue sample	Metastasis	77 (85)
	Primary	13 (15)
Metastatic site	Lymph node	54 (70)
	Including SLN	*32 (60)*
	Sub-cutaneous	14 (18)
	Pulmonary	7 (9)
	Other	2 (3)
Percentage of tumor cells (TC)	≤1%	6 (7)
	1 < TC < 10%	12 (13)
	10 ≤ TC < 50%	15 (17)
	≥50	57 (63)
Mean percentage of tumor cell in SLN	9.3%	
Molecular analysis	Assessed	90 (100)
	Not contributive	5 (5)
Molecular status	mutated *BRAF*	28 (33)
	*BRAF V600E*	22 (79)
	*BRAF V600K*	6 (21)
	*wt-BRAF*	57 (67)
IHC analysis	Assessed	90 (100)
	Not contributive	2 (2)
IHC status	*BRAF V600E*	29 (33)
	wt-*BRAF V600E*	59 (67)
**(B)**		
**Pathological Characteristics of Metastatic SLN**		**N (%)**
		**90 (100)**
SLN	Number	32 (35)
SLN tumor size	<0.1 mm	1 (3)
	0.1–1 mm	14 (43)
	>1 mm	17 (53)
SLN tumor microanatomical localization	Sub-capsular	8 (25)
	Parenchymal	6 (19)
	Combined	10 (31)
	Multifocal	6 (19)
	Extensive	2 (6)

**Table 3:** Analysis of discordant results.

	Cases	Diagnostic	Location	Stage	TC %	Idylla	Idylla Explore	ΔCQ (Idylla)	*BRAF* VE1 IHC	Other Sample Available	TC (%)	Molecular Methods	Results
Positive Idylla/Negative IHC	#62	Metastatic melanoma	Sub-cutaneous	IV	20	*V600E/E2/D*	Amplification	6.54	Negative	Metastatic melanoma	20	Idylla and NGS and IHC	Wild type
Negative Idylla/Positive IHC	#2	Metastatic melanoma	Sentinel lymph node	IIIA	5	Wild type	Delayed amplification	10.07	Positive	Metastatic melanoma	50	PS **	*V600E*
	#17	Metastatic melanoma	Sentinel lymph node	IIIA	5	Wild type	Delayed amplification	8.69	Positive	Metastatic melanoma	80	Idylla	*V600E/E2/D*
	#22	Metastatic melanoma	Lymph node	IIIA	5	Wild type	Delayed amplification	11.39	Positive	Metastatic melanoma	70	NGS *	*V600E*
	#23	Metastatic melanoma	Sentinel lymph node	IIIA	≤1	Wild type	No amplification	Not applicable	Positive	Primitive melanoma	30	Idylla	*V600E/E2/D*
	#27	Metastatic melanoma	Sentinel lymph node	IIIA	1 < CT < 5	Wild type	Delayed amplification	10.08	Positive	Metastatic melanoma	50	PS **	*V600E*
	#28	Metastatic melanoma	Sentinel lymph node	IIIA	≤1	Wild type	Delayed amplification	9.9	Positive	Metastatic melanoma	80	PS **	*V600E*

Abbreviations: NGS: Next generation sequencing; PS: pyrosequencing; * NGS Ion GeneStudio™ S5 Thermo Fisher Scientific, Illkirch-Graffenstaden, France—Ion AmpliSeq™ Cancer Hotspot Panel V2); ** Pyrosequencing (Qiagen, Hilden, Germany—Therascreen *BRAF* Pyro Kit).

**Table 4:** Contribution of the Idylla Explore tool for cases at risk of false negative results.

Cases	SLN	Stage	% TC	Idylla Result	Delayed Amplification	Idylla Explore Tool (ΔCQ)	IHC *BRAF*	Re-Test/Other Sample
#3	yes	IIIA	1	Wild type	No	Not applicable	Negative	Not applicable
#5	yes	IIIA	<1	Wild type	Yes	ΔCQ = 10.08	Negative	Wild type (primitive)
#10	yes	IIIA	<1	Wild type	No	Not applicable	Negative	Wild type (metastasis)
#11	yes	IIIA	<1	Wild type	Yes	ΔCQ = 10.98	Negative	Not applicable
#23	yes	IIIA	<1	Wild type	No	Not applicable	Positive	*BRAF V600E* (primitif)
#28	yes	IIIA	<1	Wild type	Yes	ΔCQ = 9.90	Positive	*BRAF V600E* (metastasis)

**Table 5:** Literature review of studies on the performance of Idylla for *BRAF* detection in melanoma.

Ref.	Study	Gene	Idylla Test	CE-IVD	Mutation Detected for BRAF	Sample	Number of Samples	Type of Tumor	Sample Origin	TAT min	Reference Method	Concordance for BRAF %	Sens %	Spe %	PPV %	NPV %
Melchior et al. 2015 [25]	Multicenter retrospective	*BRAF*	Idylla *BRAF* Mutation Test	CE-IVD	*V600E/E2/D V600K/R/M*	FFPE tissue	139	Melanoma	*NS*	90	SS, RT-PCR, ddPCR, HRM	97.84	*NS*	*NS*	*NS*	*NS*
Janku et al. 2015 [16]	Retrospective	*BRAF*	Idylla *BRAF* Mutation Test	*NS*	*V600E/E2/D V600K/R/M*	FFPE tissue	60	Melanoma, CRC, PTC and others	*NS*	90	RT-PCR, NGS	97 (RT-PCR) 100 (NGS)	95	97	98	92
Janku et al. 2016 [26]	Prospective	*BRAF*	*BRAF* Mutation Test prototype	RUO	Not specified	Cell-free DNA vs FFPE	160	CRC, Melanoma, NSCLCC and others	Blood	90	PCR-based method, mass spectrometry, NGS	88	73	98	96	85
Schiefer et al. 2016 [17]	Multicenter Retrospective	*BRAF*	Idylla *BRAF* Mutation test	CE-IVD	*V600E/E2/D V600K/R/M*	FFPE tissue	419	Melanoma, PTC, CRC and others	Primary and Metastases	90	SS, PS, NGS	96.2 (SS); 97.5 (PS)	*NS*	*NS*	*NS*	*NS*
Harlé et al. 2016 [27]	Retrospective	*BRAF*	Idylla *BRAF* Mutation Test	CE-IVD	*V600E/E2/D V600K/R/M*	FFPE tissue	59	Melanoma	*NS*	90	HRM, real-time PCR, NGS, IHC	*NS*	93.5 (NGS)	100	100	93.3
Barel et al. 2018 [28]	Retrospective	*NRAS-BRAF-EGFR*	Idylla NRAS-*BRAF*-EGFR S492R Mutation Test	RUO	*V600 E/E2/D V600 K/R*	FFPE tissue	36	Melanoma	Primary and metastases	110	NGS, IHC	97.2 (overall)	*NS*	*NS*	*NS*	*NS*
Bisschop et al. 2018 [29]	Retrospective	*BRAF*	Idylla *BRAF* Mutation Test	CE-IVD	*V600E/E2/D V600K/R/M*	FFPE tissue	37	Melanoma	Primary and metastases	90	HRM, SS, IHC, NGS	97.3 (overall)	100	0.94	100	100
Long-Mira et al. 2018 [30]	Prospective	*BRAF—NRAS*	ctNRAS-*BRAF* Mutation Test	RUO	*V600E/E2/D V600 K/R/M*	Cell-free DNA vs FFPE	19	Melanoma	Blood	90	PS, NGS	84	80	89	*NS*	*NS*
Seremet et al. 2018 [31]	Prospective short communication	*BRAF-NRAS*	*NS*	*NS*	*NS*	cell-free DNA	7	Melanoma	Blood	*NS*	No	*NS*	*NS*	*NS*	*NS*	*NS*
Serre et al. 2018 [32]	Prospective and retrospective	*BRAF*	Idylla *BRAF* Mutation Test	CE-IVD	*V600E/E2/D V600K/R/M*	FFPE tissue	37	Melanoma	Metastases	*90*	No	*NS*	*NS*	*NS*	*NS*	*NS*
Vallée et al. 2019 [33]	Prospective	*NRAS-BRAF-EGFR*	Idylla NRAS-*BRAF*-EGFR S492R Mutation Test	RUO	*V600 E/E2/D* *V600 K/R*	FFPE tissue	65	Melanoma	Primary and metastases	120	IHC, ASA, SS, ddPCR	92.1 (overall)	100	100	100	100
Huang et al. 2019 [34]	Retrospective	*BRAF*	Idylla NRAS-*BRAF* and Idylla *BRAF* Mutation Test	*NS*	*V600 E/E2/D V600 K/R*	FFPE tissue	210	CRC, Melanoma, NSCLCC and others	*NS*	*NS*	NGS, SS	100	*NS*	*NS*	*NS*	*NS*
Bourhis et al. 2019 [35]	Retrospective	*BRAF*	Idylla *BRAF* Mutation Test	CE-IVD	*V600E/E2/D V600K/R/M*	FFPE tissue and decalcified tissue	11 samples (paired)	Melanoma, Hairy cell leukemia	Metastases	90	IHC	100 (except decalcified samples)	*NS*	*NS*	*NS*	*NS*
Van Haele et al. 2020 [36]	Prospective	*BRAF*	Idylla *BRAF* Mutation Test	CE-IVD	*V600E/E2/D V600K/R/M*	FFPE tissue and cell block	48	Melanoma, NSCLCC, CRC	Metastases	90	NGS, Cobas	100 (NGS)	*NS*	*NS*	*NS*	*NS*
Petty et al. 2020 [37]	Retrospective	*BRAF*	Idylla BRAF Mutation Test	*NS*	*V600E/E2/D V600K/R/M*	FFPE tissue and cell block	23	Melanoma	Primary and metastases	90	SS, ARMS	100	100	100	100	100
Colombino et al. 2020 [38]	Retrospective	*BRAF*	*NS*	*NS*	*V600E/E2/D V600K/R/M*	DNA	319	Melanoma	Primary and metastases	120	SS, PS, NGS	98.4 (*BRAF*+)	*NS*	*NS*	*NS*	*NS*

**Table 6:** Overview of studies combining immunohistochemistry and Idylla for *BRAF* evaluation.

Reference	Tumor Type	Number of Samples	Type Antibody	*BRAF* Mutation	CE-IVD	Immunostaining System	Sample	Idylla Method	*BRAF* Mutation with Idylla	Concordance Rate (%)	IHC Sensitivity (%)	IHC Specificity (%)	PPV (%)	NPV (%)
Durślewicz et al. 2020 [39]	CNS tumor	22	Clone VE1 (Ventana Medical System)	*BRAF V600E*	Yes	Ventana BenchMark ULTRA stainer	FFPE tissue	Idylla *BRAF* mutation Test	*V600E/E2/D V600K/R/M*	86	*NS*	*NS*	*NS*	*NS*
Sadlecki et al. 2017 [40]	Ovarian tumor	42	Clone VE1 (Ventana Medical System)	*BRAF V600E*	Yes	Ventana BenchMark GX	FFPE tissue	Idylla *BRAF* mutation Test	*V600E/E2/D V600K/R/M*	100	*NS*	*NS*	*NS*	*NS*
Bourhis et al. 2019 [35]	Metastatic melanoma and hairy cell leukemia	11	Clone VE1	*BRAF V600E*	No	Ventana Benchmark XT	FFPE tissue and decalcified	Idylla *BRAF* mutation Test	*V600E/E2/D V600K/R/M*	100	*NS*	*NS*	*NS*	*NS*
Bisschop et al. 2018 [29]	Metastatic melanoma	37	Clone VE1 (Ventana Medical System)	*BRAF V600E*	Yes	Ventana BenchMark ULTRA stainer	FFPE tissue	Idylla NRAS-*BRAF*-EGFR S492R Mutation Test	*V600 E/E2/D V600 K/R*	97.3 (overall)	94	95	*NS*	*NS*
Barel et al. 2018 [28]	Melanoma (metastatic and primary)	36	Clone VE1	*BRAF V600E*	No	Ventana Benchmark XT	FFPE tissue	Idylla NRAS-*BRAF*-EGFR S492R Mutation Test	*V600 E/E2/D V600 K/R*	100	*NS*	*NS*	*NS*	*NS*
Colling et al. 2017 [41]	CRC	20	Clone VE1 (Ventana Medical System)	*BRAF V600E*	Yes	Ventana Benchmark Immunostainer	FFPE tissue	Idylla NRAS-*BRAF*-EGFR S492R Mutation Test	*V600 E/E2/D V600 K/R*	90	*NS*	*NS*	*NS*	*NS*
Vallée et al. 2019 [33]	Melanoma (metastatic and primary)	65	Clone VE1 (Eurobio)	*BRAF V600E*	No	*NS*	FFPE tissue	Idylla NRAS-*BRAF*-EGFR S492R Mutation Test	*V600 E/E2/D V600 K/R*	89 (overall)	82,3	100	100	93
Bodnar et al. 2017 [42]	Salivary gland tumor	95	Clone VE1 (Ventana Medical System)	*BRAF V600E*	Yes	Ventana BenchMark GX	FFPE tissue	Idylla *BRAF* Mutation Test	*V600E/E2/D V600K/R/M*	97	*NS*	*NS*	*NS*	*NS*
Cardus et al. 2019 [43]	Hairy cell leukemia and B/T cell neoplasm	218	Clone VE1 (Ventana Medical System)	*BRAF V600E*	Yes	Ventana BenchMark ULTRA stainer	FFPE tissue and decalcified	Idylla *BRAF* Mutation Test	*V600E/E2/D V600K/R/M*	100	*NS*	*NS*	*NS*	*NS*

**Table 7:** Comparison of Idylla and IHC for the detection of the *BRAF V600E* mutation in melanomas.

	Idylla (Biocartis, Belgium)	IHC *BRAFV600E* (Clone VE1, Roche Ventana)
Principles of the Technology	DNA, RT-PCR	Protein expression, Antigen-Antibody
Mutations	Detection of a Group of Mutant Only *V600E/E2/D*; *V600K/R/M*	*V600E*
Cost/Patient *	140 €	54 €
Duration run	90 mn	255 mn
Hands-on time	20 mn Including block selection, cutting section or macrodissection, insertion in the cartridge	70 mn Including cutting slide, drying time, preparation of the instrument and mounting of the slide
Total duration time	110 mn	325 mn
Competence of the operator	Not required	Trained technician
Ease of interpretation	Very easy—No specific skills	Easy—Trained Pathologist
Analytical sensibility	Very high (1%)	Very high (single cell-level resolution)
Minimal amount of material	50% tumor cell and 250 mm^3^ of tissue are recommended	Few cells, methods independant of the percentage of tumor cell
Preanalytic parameter	Robust (formalin fixative)	Delicate (formalin fixative, cold ischemia)
Major advantage	Easy to use	Single cell-level
Major limitation	Impossibility to collect DNA from the cartridge after test completion for NGS	Limited to the detection of the *BRAF V600E* mutant protein

* In our laboratory.

## Data Availability

Data are available by request to the corresponding author.

## References

[1] Sung H., Ferlay J., Siegel R.L., Laversanne M., Soerjomataram I., Jemal A., Bray F. (2021). Global Cancer Statistics 2020: GLOBOCAN Estimates of Incidence and Mortality Worldwide for 36 Cancers in 185 Countries. CA Cancer J. Clin..

[2] Siegel R.L., Miller K.D., Fuchs H.E., Jemal A. (2022). Cancer Statistics, 2022. CA Cancer J. Clin..

[3] Garbe C., Amaral T., Peris K., Hauschild A., Arenberger P., Bastholt L., Bataille V., Del Marmol V., Dréno B., Fargnoli M.C. (2020). European Consensus-Based Interdisciplinary Guideline for Melanoma. Part 1: Diagnostics—Update 2019. Eur. J. Cancer.

[4] Prieto V.G. (2017). Sentinel Lymph Nodes in Cutaneous Melanoma. Clin. Lab. Med..

[5] Garbe C., Amaral T., Peris K., Hauschild A., Arenberger P., Bastholt L., Bataille V., Del Marmol V., Dréno B., Fargnoli M.C. (2020). European Consensus-Based Interdisciplinary Guideline for Melanoma. Part 2: Treatment—Update 2019. Eur. J. Cancer.

[6] Luke J.J., Flaherty K.T., Ribas A., Long G.V. (2017). Targeted Agents and Immunotherapies: Optimizing Outcomes in Melanoma. Nat. Rev. Clin. Oncol..

[7] Davies H., Bignell G.R., Cox C., Stephens P., Edkins S., Clegg S., Teague J., Woffendin H., Garnett M.J., Bottomley W. (2002). Mutations of the BRAF Gene in Human Cancer. Nature.

[8] Platz A., Egyhazi S., Ringborg U., Hansson J. (2008). Human Cutaneous Melanoma; a Review of NRAS and BRAF Mutation Frequencies in Relation to Histogenetic Subclass and Body Site. Mol. Oncol..

[9] Bergdorf K.N., Lee L.A., Weiss V.L. (2020). BRAF Molecular Testing in Cytopathology: Implications for Diagnosis, Prognosis, and Targeted Therapeutics. Cancer Cytopathol..

[10] Cheng L., Lopez-Beltran A., Massari F., MacLennan G.T., Montironi R. (2018). Molecular Testing for BRAF Mutations to Inform Melanoma Treatment Decisions: A Move toward Precision Medicine. Mod. Pathol..

[11] Long G.V., Wilmott J.S., Capper D., Preusser M., Zhang Y.E., Thompson J.F., Kefford R.F., von Deimling A., Scolyer R.A. (2013). Immunohistochemistry Is Highly Sensitive and Specific for the Detection of V600E BRAF Mutation in Melanoma. Am. J. Surg. Pathol..

[12] Ritterhouse L.L., Barletta J.A. (2015). BRAF V600E Mutation-Specific Antibody: A Review. Semin. Diagn. Pathol..

[13] Long E., Ilie M., Lassalle S., Butori C., Poissonnet G., Washetine K., Mouroux J., Lespinet V., Lacour J.P., Taly V. (2015). Why and How Immunohistochemistry Should Now Be Used to Screen for the BRAFV600E Status in Metastatic Melanoma? The Experience of a Single Institution (LCEP, Nice, France). J. Eur. Acad. Dermatol. Venereol..

[14] Long-Mira E., Washetine K., Hofman P. (2016). Sense and Nonsense in the Process of Accreditation of a Pathology Laboratory. Virchows Arch..

[15] Lhermitte B., Egele C., Weingertner N., Ambrosetti D., Dadone B., Kubiniek V., Burel-Vandenbos F., Coyne J., Michiels J.-F., Chenard M.-P. (2017). Adequately Defining Tumor Cell Proportion in Tissue Samples for Molecular Testing Improves Interobserver Reproducibility of Its Assessment. Virchows Arch..

[16] Janku F., Claes B., Huang H.J., Falchook G.S., Devogelaere B., Kockx M., Bempt I.V., Reijans M., Naing A., Fu S. (2015). BRAF Mutation Testing with a Rapid, Fully Integrated Molecular Diagnostics System. Oncotarget.

[17] Schiefer A.-I., Parlow L., Gabler L., Mesteri I., Koperek O., von Deimling A., Streubel B., Preusser M., Lehmann A., Kellner U. (2016). Multicenter Evaluation of a Novel Automated Rapid Detection System of BRAF Status in Formalin-Fixed, Paraffin-Embedded Tissues. J. Mol. Diagn..

[18] Ilie M.I., Lassalle S., Long-Mira E., Bonnetaud C., Bordone O., Lespinet V., Lamy A., Sabourin J.-C., Haudebourg J., Butori C. (2014). Diagnostic Value of Immunohistochemistry for the Detection of the BRAF(V600E) Mutation in Papillary Thyroid Carcinoma: Comparative Analysis with Three DNA-Based Assays. Thyroid.

[19] Van Akkooi A.C.J., De Wilt J.H.W., Verhoef C., Graveland W.J., Van Geel A.N., Kliffen M., Eggermont A.M.M. (2006). High Positive Sentinel Node Identification Rate by EORTC Melanoma Group Protocol. Prognostic Indicators of Metastatic Patterns after Sentinel Node Biopsy in Melanoma. Eur. J. Cancer.

[20] Dewar D.J., Newell B., Green M.A., Topping A.P., Powell B.W.E.M., Cook M.G. (2004). The Microanatomic Location of Metastatic Melanoma in Sentinel Lymph Nodes Predicts Nonsentinel Lymph Node Involvement. J. Clin. Oncol..

[21] Lito P., Rosen N., Solit D.B. (2013). Tumor Adaptation and Resistance to RAF Inhibitors. Nat. Med..

[22] Yancovitz M., Litterman A., Yoon J., Ng E., Shapiro R.L., Berman R.S., Pavlick A.C., Darvishian F., Christos P., Mazumdar M. (2012). Intra- and Inter-Tumor Heterogeneity of BRAF(V600E)) Mutations in Primary and Metastatic Melanoma. PLoS ONE.

[23] Satzger I., Marks L., Kerick M., Klages S., Berking C., Herbst R., Völker B., Schacht V., Timmermann B., Gutzmer R. (2015). Allele Frequencies of BRAFV600 Mutations in Primary Melanomas and Matched Metastases and Their Relevance for BRAF Inhibitor Therapy in Metastatic Melanoma. Oncotarget.

[24] Hodis E., Watson I.R., Kryukov G.V., Arold S.T., Imielinski M., Theurillat J.-P., Nickerson E., Auclair D., Li L., Place C. (2012). A Landscape of Driver Mutations in Melanoma. Cell.

[25] Melchior L., Grauslund M., Bellosillo B., Montagut C., Torres E., Moragón E., Micalessi I., Frans J., Noten V., Bourgain C. (2015). Multi-Center Evaluation of the Novel Fully-Automated PCR-Based Idylla^TM^ BRAF Mutation Test on Formalin-Fixed Paraffin-Embedded Tissue of Malignant Melanoma. Exp. Mol. Pathol..

[26] Janku F., Huang H.J., Claes B., Falchook G.S., Fu S., Hong D., Ramzanali N.M., Nitti G., Cabrilo G., Tsimberidou A.M. (2016). BRAF Mutation Testing in Cell-Free DNA from the Plasma of Patients with Advanced Cancers Using a Rapid, Automated Molecular Diagnostics System. Mol. Cancer Ther..

[27] Harlé A., Salleron J., Franczak C., Dubois C., Filhine-Tressarieu P., Leroux A., Merlin J.-L. (2016). Detection of BRAF Mutations Using a Fully Automated Platform and Comparison with High Resolution Melting, Real-Time Allele Specific Amplification, Immunohistochemistry and Next Generation Sequencing Assays, for Patients with Metastatic Melanoma. PLoS ONE.

[28] Barel F., Guibourg B., Lambros L., Flahec G., Marcorelles P., Uguen A. (2018). Evaluation of a Rapid, Fully Automated Platform for Detection of BRAF and NRAS Mutations in Melanoma. Acta Derm. Venerol..

[29] Bisschop C., Ter Elst A., Bosman L.J., Platteel I., Jalving M., Van den Berg A., Diepstra A., Van Hemel B., Diercks G.F.H., Hospers G.A.P. (2018). Rapid BRAF Mutation Tests in Patients with Advanced Melanoma: Comparison of Immunohistochemistry, Droplet Digital PCR, and the Idylla Mutation Platform. Melanoma Res..

[30] Long-Mira E., Ilie M., Chamorey E., Leduff-Blanc F., Montaudié H., Tanga V., Allégra M., Lespinet-Fabre V., Bordone O., Bonnetaud C. (2018). Monitoring BRAF and NRAS Mutations with Cell-Free Circulating Tumor DNA from Metastatic Melanoma Patients. Oncotarget.

[31] Seremet T., Planken S., Schreuer M., Jansen Y., Delaunoy M., El Housni H., Lienard D., Del Marmol V., Heimann P., Neyns B. (2018). Illustrative Cases for Monitoring by Quantitative Analysis of BRAF/NRAS CtDNA Mutations in Liquid Biopsies of Metastatic Melanoma Patients Who Gained Clinical Benefits from Anti-PD1 Antibody Therapy. Melanoma Res..

[32] Serre D., Salleron J., Husson M., Leroux A., Gilson P., Merlin J.-L., Geoffrois L., Harlé A. (2018). Accelerated BRAF Mutation Analysis Using a Fully Automated PCR Platform Improves the Management of Patients with Metastatic Melanoma. Oncotarget.

[33] Vallée A., Denis-Musquer M., Herbreteau G., Théoleyre S., Bossard C., Denis M.G. (2019). Prospective Evaluation of Two Screening Methods for Molecular Testing of Metastatic Melanoma: Diagnostic Performance of BRAF V600E Immunohistochemistry and of a NRAS-BRAF Fully Automated Real-Time PCR-Based Assay. PLoS ONE.

[34] Huang H., Springborn S., Haug K., Bartow K., Samra H., Menon S., Mackinnon A.C. (2019). Evaluation, Validation, and Implementation of the Idylla System as Rapid Molecular Testing for Precision Medicine. J. Mol. Diagn..

[35] Bourhis A., Le Flahec G., Uguen A. (2019). Decalcification Can Cause the Failure of BRAF Molecular Analyses and Anti-BRAFV600E VE1 Immunohistochemistry. Pathol. Int..

[36] Van Haele M., Vander Borght S., Ceulemans A., Wieërs M., Metsu S., Sagaert X., Weynand B. (2020). Rapid Clinical Mutational Testing of KRAS, BRAF and EGFR: A Prospective Comparative Analysis of the Idylla Technique with High-Throughput next-Generation Sequencing. J. Clin. Pathol..

[37] Petty D.R., Hassan O.A., Barker C.S., O’Neill S.S. (2020). Rapid BRAF Mutation Testing in Pigmented Melanomas. Am. J. Dermatopathol..

[38] Colombino M., Rozzo C., Paliogiannis P., Casula M., Manca A., Doneddu V., Fedeli M.A., Sini M.C., Palomba G., Pisano M. (2020). Comparison of BRAF Mutation Screening Strategies in a Large Real-Life Series of Advanced Melanoma Patients. J. Clin. Med..

[39] Durślewicz J., Klimaszewska-Wiśniewska A., Antosik P., Kasperska A., Grzanka D., Szylberg T., Szylberg Ł. (2020). Detection of BRAF V600E Mutation in Ganglioglioma and Pilocytic Astrocytoma by Immunohistochemistry and Real-Time PCR-Based Idylla Test. Dis. Markers.

[40] Sadlecki P., Walentowicz P., Bodnar M., Marszalek A., Grabiec M., Walentowicz-Sadlecka M. (2017). Determination of BRAF V600E (VE1) Protein Expression and BRAF Gene Mutation Status in Codon 600 in Borderline and Low-Grade Ovarian Cancers. Tumour Biol..

[41] Colling R., Wang L.M., Soilleux E. (2017). Validating a Fully Automated Real-Time PCR-Based System for Use in the Molecular Diagnostic Analysis of Colorectal Carcinoma: A Comparison with NGS and IHC. J. Clin. Pathol..

[42] Bodnar M., Burduk P., Antosik P., Jarmuz-Szymczak M., Wierzbicka M., Marszalek A. (2017). Assessment of BRAF V600E (VE1) Protein Expression and BRAF Gene Mutation Status in Codon 600 in Benign and Malignant Salivary Gland Neoplasms. J. Oral. Pathol. Med..

[43] Cardus B., Colling R., Hamblin A., Soilleux E. (2019). Comparison of Methodologies for the Detection of BRAF Mutations in Bone Marrow Trephine Specimens. J. Clin. Pathol..

[44] Viray H., Li K., Long T.A., Vasalos P., Bridge J.A., Jennings L.J., Halling K.C., Hameed M., Rimm D.L. (2013). A Prospective, Multi-Institutional Diagnostic Trial to Determine Pathologist Accuracy in Estimation of Percentage of Malignant Cells. Arch. Pathol. Lab. Med..

[45] Smits A.J.J., Kummer J.A., Dde Bruin P.C., Bol M., Van den Tweel J.G., Seldenrijk K.A., Willems S.M., Offerhaus G.J.A., De Weger R.A., Van Diest P.J. (2014). The Estimation of Tumor Cell Percentage for Molecular Testing by Pathologists Is Not Accurate. Mod. Pathol..

[46] Chen G., Dudley J., Tseng L.-H., Smith K., Gurda G.T., Gocke C.D., Eshleman J.R., Lin M.-T. (2015). Lymph Node Metastases of Melanoma: Challenges for BRAF Mutation Detection. Hum. Pathol..

[47] Dudley J.C., Gurda G.T., Tseng L.-H., Anderson D.A., Chen G., Taube J.M., Gocke C.D., Eshleman J.R., Lin M.-T. (2014). Tumor Cellularity as a Quality Assurance Measure for Accurate Clinical Detection of BRAF Mutations in Melanoma. Mol. Diagn. Ther..

[48] Long G.V., Hauschild A., Santinami M., Atkinson V., Mandalà M., Chiarion-Sileni V., Larkin J., Nyakas M., Dutriaux C., Haydon A. (2017). Adjuvant Dabrafenib plus Trametinib in Stage III BRAF-Mutated Melanoma. N. Engl. J. Med..

[49] Maio M., Lewis K., Demidov L., Mandalà M., Bondarenko I., Ascierto P.A., Herbert C., Mackiewicz A., Rutkowski P., Guminski A. (2018). Adjuvant Vemurafenib in Resected, BRAFV600 Mutation-Positive Melanoma (BRIM8): A Randomised, Double-Blind, Placebo-Controlled, Multicentre, Phase 3 Trial. Lancet Oncol..

[50] Varada S., Mahalingam M. (2015). Mutation Stability in Primary and Metastatic Melanoma: What We Know and What We Don’t. Histol. Histopathol..

[51] Ito T., Tanaka Y., Murata M., Kaku-Ito Y., Furue K., Furue M. (2021). BRAF Heterogeneity in Melanoma. Curr. Treat. Options Oncol..

[52] Chang G.A., Wiggins J.M., Corless B.C., Syeda M.M., Tadepalli J.S., Blake S., Fleming N., Darvishian F., Pavlick A., Berman R. (2020). TERT, BRAF, and NRAS Mutational Heterogeneity between Paired Primary and Metastatic Melanoma Tumors. J. Investig. Dermatol..

[53] Heinzerling L., Baiter M., Kühnapfel S., Schuler G., Keikavoussi P., Agaimy A., Kiesewetter F., Hartmann A., Schneider-Stock R. (2013). Mutation Landscape in Melanoma Patients Clinical Implications of Heterogeneity of BRAF Mutations. Br. J. Cancer.

[54] Manfredi L., Meyer N., Tournier E., Grand D., Uro-Coste E., Rochaix P., Brousset P., Lamant L. (2016). Highly Concordant Results Between Immunohistochemistry and Molecular Testing of Mutated V600E BRAF in Primary and Metastatic Melanoma. Acta Derm. Venereol..

[55] Guadarrama-Orozco J.A., Ortega-Gómez A., Ruiz-García E.B., Astudillo-de la Vega H., Meneses-García A., Lopez-Camarillo C. (2016). Braf V600E Mutation in Melanoma: Translational Current Scenario. Clin. Transl. Oncol..

[56] Cormican D., Kennedy C., Murphy S., Werner R., Power D.G., Heffron C.C.B.B. (2019). High Concordance of BRAF Mutational Status in Matched Primary and Metastatic Melanoma. J. Cutan. Pathol..

[57] Nielsen L.B., Dabrosin N., Sloth K., Bønnelykke-Behrndtz M.L., Steiniche T., Lade-Keller J. (2018). Concordance in BRAF V600E Status over Time in Malignant Melanoma and Corresponding Metastases. Histopathology.

[58] Parakh S., Murphy C., Lau D., Cebon J.S., Andrews M.C. (2015). Response to MAPK Pathway Inhibitors in BRAF V600M-Mutated Metastatic Melanoma. J. Clin. Pharm. Ther..

[59] Popescu A., Haidar A., Anghel R.M. (2018). Treating Malignant Melanoma When a Rare BRAF V600M Mutation Is Present: Case Report and Literature Review. Rom. J. Intern. Med..

[60] Eckhart L., Bach J., Ban J., Tschachler E. (2000). Melanin Binds Reversibly to Thermostable DNA Polymerase and Inhibits Its Activity. Biochem. Biophys. Res. Commun..

[61] Cook M.G., Green M.A., Anderson B., Eggermont A.M.M., Ruiter D.J., Spatz A., Kissin M.W., Powell B.W.E.M., EORTC Melanoma Group (2003). The Development of Optimal Pathological Assessment of Sentinel Lymph Nodes for Melanoma. J. Pathol..

[62] Cook M.G., Massi D., Szumera-Ciećkiewicz A., Van den Oord J., Blokx W., Van Kempen L.C., Balamurugan T., Bosisio F., Koljenović S., Portelli F. (2019). An Updated European Organisation for Research and Treatment of Cancer (EORTC) Protocol for Pathological Evaluation of Sentinel Lymph Nodes for Melanoma. Eur. J. Cancer.

[63] Engel K.B., Moore H.M. (2011). Effects of Preanalytical Variables on the Detection of Proteins by Immunohistochemistry in Formalin-Fixed, Paraffin-Embedded Tissue. Arch. Pathol. Lab. Med..

[64] Menzies A.M., Lum T., Wilmott J.S., Hyman J., Kefford R.F., Thompson J.F., O’Toole S., Long G.V., Scolyer R.A. (2014). Intrapatient Homogeneity of BRAFV600E Expression in Melanoma. Am. J. Surg. Pathol..

[65] Dummer R., Hauschild A., Santinami M., Atkinson V., Mandalà M., Kirkwood J.M., Chiarion Sileni V., Larkin J., Nyakas M., Dutriaux C. (2020). Five-Year Analysis of Adjuvant Dabrafenib plus Trametinib in Stage III Melanoma. N. Engl. J. Med..

[66] Dummer R., Lebbé C., Atkinson V., Mandalà M., Nathan P.D., Arance A., Richtig E., Yamazaki N., Robert C., Schadendorf D. (2020). Combined PD-1, BRAF and MEK Inhibition in Advanced BRAF-Mutant Melanoma: Safety Run-in and Biomarker Cohorts of COMBI-i. Nat. Med..

[67] Hauschild A., Dummer R., Schadendorf D., Santinami M., Atkinson V., Mandalà M., Chiarion-Sileni V., Larkin J., Nyakas M., Dutriaux C. (2018). Longer Follow-Up Confirms Relapse-Free Survival Benefit with Adjuvant Dabrafenib Plus Trametinib in Patients with Resected BRAF V600-Mutant Stage III Melanoma. J. Clin. Oncol..

[68] Bellomo D., Arias-Mejias S.M., Ramana C., Heim J.B., Quattrocchi E., Sominidi-Damodaran S., Bridges A.G., Lehman J.S., Hieken T.J., Jakub J.W. (2020). Model Combining Tumor Molecular and Clinicopathologic Risk Factors Predicts Sentinel Lymph Node Metastasis in Primary Cutaneous Melanoma. JCO Precis. Oncol..

[69] Yousaf A., Tjien-Fooh F.J., Rentroia-Pacheco B., Quattrocchi E., Kobic A., Tempel D., Kolodney M., Meves A. (2021). Validation of CP-GEP (Merlin Assay) for Predicting Sentinel Lymph Node Metastasis in Primary Cutaneous Melanoma Patients: A U.S. Cohort Study. Int. J. Dermatol..

[70] Salvianti F., Massi D., De Giorgi V., Gori A., Pazzagli M., Pinzani P. (2019). Evaluation of the Liquid Biopsy for the Detection of BRAFV600E Mutation in Metastatic Melanoma Patients. Cancer Biomark..

